# Accuracy of photon-counting detector CT-based iodine maps for myocardial late enhancement detection

**DOI:** 10.1007/s00330-025-11622-0

**Published:** 2025-05-01

**Authors:** Giuseppe Tremamunno, Akos Varga-Szemes, Dmitrij Kravchenko, Andrea Laghi, Fabian Bamberg, Moritz C. Halfmann, Pál Spruill Suranyi, Milán Vecsey-Nagy, Tilman Emrich, Muhammad Taha Hagar

**Affiliations:** 1 Department of Radiology and Radiological Science, Medical University of South Carolina, Charleston, SC USA; 2 Radiology Unit, Department of Medical Surgical Sciences and Translational Medicine, Sapienza University of Rome, Sant’Andrea University Hospital, Rome, Italy; 3 Department of Diagnostic and Interventional Radiology, University Hospital Bonn, Bonn, Germany; 4 Department of Diagnostic and Interventional Radiology, Medical Center, Faculty of Medicine, University of Freiburg, Freiburg, Germany; 5 Department of Diagnostic and Interventional Radiology, University Medical Center of the Johannes Gutenberg-University, Mainz, Germany; 6 Heart and Vascular Center, Semmelweis University, Budapest, Hungary; 7 German Centre for Cardiovascular Research, Partner site Rhine-Main, Mainz, Germany

**Keywords:** PCD-CT, Cardiac MRI, Cardiac CT, Iodine, Late enhancement

## Abstract

**Objectives:**

To evaluate the diagnostic accuracy of iodine maps from photon-counting detector (PCD) CT in detecting myocardial late enhancement compared to late gadolinium enhancement (LGE)-MRI.

**Materials and methods:**

In this retrospective analysis of a prospective cohort, patients underwent cardiac MRI with LGE followed by late iodine enhancement (LIE)-CT using dual-source PCD-CT. LIE-CT was performed 5 min post-intravenous administration of 100 mL iopromide (370 mg I/mL) using an ECG-triggered sequential protocol with full spectral capabilities (120 kVp, 144 × 0.4 mm collimation). Iodine maps were reconstructed with a quantitative kernel (Qr40) and iterative reconstruction. Two radiologists independently rated image quality on a four-point scale (1: “poor” to 4: “excellent”). Diagnostic performance was assessed per-patient and per-segment using LGE-MRI as reference, and inter-reader agreement was analyzed using Cohen’s kappa (κ).

**Results:**

The study included 27 patients (52% female; mean age 52.9 ± 17.2 years). Twelve (44%) had positive LGE, with 87/459 (19%) myocardial segments affected. Image quality was rated as good, with no significant differences between readers (median 3 [2–4] vs 3 [3–4]; *p* = 0.058). Per-patient sensitivities were 100% and 91.7%, specificities 73.3% and 80.0%, and accuracies 85.2%, respectively. Per-segment sensitivities, specificities, and accuracies were 74.7%, 94.9%, and 91.1% (reader 1) and 66.7%, 96.4%, and 90.7% (reader 2). Substantial inter-reader agreement was observed (κ = 0.70 per patient, 0.63 per segment).

**Conclusion:**

Iodine maps from PCD-CT demonstrate high diagnostic accuracy for assessing myocardial late enhancement, with substantial inter-reader agreement. These findings suggest that PCD-CT may serve as a valuable alternative to LGE-MRI.

**Key Points:**

***Question*** *Can PCD CT iodine maps detect myocardial late enhancement with accuracy comparable to LGE-MRI*?

***Findings*** *PCD-CT iodine maps achieved high accuracy (85.2% per patient, > 90% per segment) with substantial inter-reader agreement*.

***Clinical relevance*** *PCD-CT iodine maps offer a valuable alternative to LGE-MRI for myocardial late enhancement assessment, especially for patients with contraindications to MRI*.

**Graphical Abstract:**

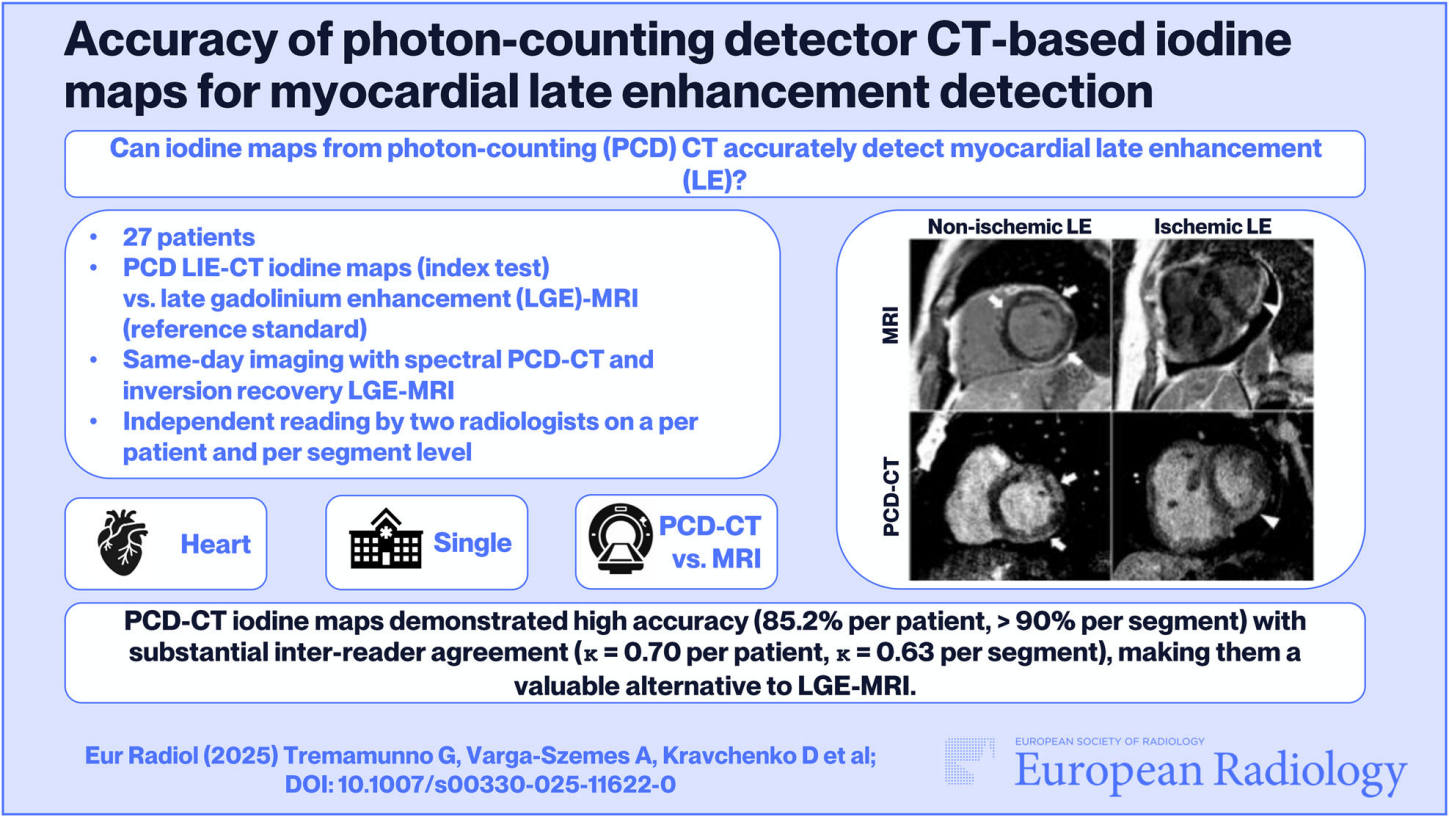

## Introduction

Late gadolinium enhancement (LGE) cardiac MRI is a well-established diagnostic and prognostic tool for various acute and chronic cardiac conditions, and is included in several clinical guidelines [1–4]. Cardiomyopathies can result in fibrotic changes of the myocardial tissue, consequently, focally expanding the extracellular volume (ECV), which can be effectively visualized by LGE [5]. In other conditions, such as myocardial infarction or myocarditis, LGE occurs due to damaged myocyte membranes, permitting contrast agent diffusion into the extracellular space [6]. LGE is primarily assessed qualitatively, where the presence and pattern of enhancement are critical for distinguishing between ischemic and non-ischemic cardiomyopathies [7–9]. However, despite its advantages, cardiac MRI has several limitations: it is time-consuming, susceptible to artifacts, contraindicated for certain implants, and requires substantial patient cooperation, which can be challenging in cases of severe claustrophobia [10].

In recent years, cardiac CT has emerged as a potential alternative for myocardial tissue characterization and fibrosis detection [11–13]. Iodine and gadolinium share similar pharmacokinetics in infarcted and non-infarcted myocardium, making late iodine enhancement (LIE) visualization on CT feasible [14]. However, CT image quality and contrast-to-noise ratios (CNR) in LIE assessment are compromised by lower tissue and contrast resolution, compared to LGE MRI [14].

Photon-counting detector CT (PCD-CT), offers the potential to refine cardiac assessment and myocardial tissue characterization [15], as image quality is improved through superior spatial resolution, elimination of electronic noise, while maintaining high temporal resolution, and spectral capabilities [16–19]. Beyond its inherent improvements in spatial resolution and spectral capabilities, PCD-CT enables advanced post-processing techniques, such as iodine maps. These iodine quantification maps derived from PCD-CT allow for ECV quantification, providing values comparable to MRI [20, 21].

These iodine maps could also be valuable for LIE assessment by mimicking the “nulling” of myocardium seen in LGE MRI, as they suppress surrounding soft tissues and highlight contrast-enhanced regions. Despite these promising developments, a dedicated analysis comparing scar detection on PCD-CT using iodine maps with the well-established standard of LGE MRI is still lacking. Therefore, the purpose of our study was to evaluate the diagnostic accuracy of iodine maps from PCD-CT in detecting myocardial LIE compared to LGE MRI, as the reference standard.

## Materials and methods

### Study population

This retrospective study builds upon a previously approved, prospective investigation conducted at our academic medical center, in compliance with the Health Insurance Portability and Accountability Act. Institutional review board approval was obtained. The original cohort, recruited between July 2021 and January 2022, consisted of consecutive participants undergoing cardiac MRI for various clinical indications, followed by a same-day PCD-CT scan [21]. Inclusion criteria required participants to be at least 18 years of age, with a clinical indication for cardiac MRI, while exclusion criteria included refusal to consent, contraindications to iodine-based contrast media, and technical issues with MRI or CT. Additional inclusion criteria of this study comprised the availability of spectral reconstructions.

### Image acquisition

#### MRI acquisition protocol

All patients underwent LGE imaging as part of a standard of care cardiac MRI examination performed on a 1.5-T system (MAGNETOM Avanto, Siemens Healthineers). The imaging protocol included standard cardiac acquisitions, however, only LGE sequences were used for the current analysis. LGE images were acquired 10–12 min after the administration of 0.1 mmol/kg body weight gadobutrol (Gadavist, Bayer Healthcare) using a balanced steady-state free-precession sequence with an inversion recovery pulse. Imaging was performed in both short- and long-axis orientations with the following parameters: repetition time/echo time, 2.6/1.1 ms; field of view, 340 × 255 mm²; section thickness, 8 mm; image acquisition matrix, 192 × 106; and reconstruction matrix, 192 × 144; resulting in an in-plane spatial resolution of 1.77 × 1.77 mm². A parallel imaging acceleration factor of 2 was applied, with Cartesian readout. These LGE images served as the reference standard for this analysis.

#### CT late enhancement (LE) acquisition protocol

All participants were scanned using a first-generation dual-source PCD-CT system (NAEOTOM Alpha; Siemens Healthineers, software version VA40) with a gantry rotation time of 0.25 s. Tube voltage was set to 120 kV, and the tube current was automatically adjusted to meet the selected image quality (CARE Dose4D, Siemens Healthineers). Initially, an unenhanced cardiac scan was performed. Subsequently, 100 mL of iodinated contrast media (iopromide, Ultravist, 370 mg I/mL; Bayer Healthcare) was administered intravenously, followed by a 20-mL saline chaser at a flow rate of 5–6 mL/s. After acquiring the coronary CT angiography, a delayed phase scan was conducted 5 min post-contrast. The full spectral mode (collimation 144 × 0.4 mm) was used, with automatic exposure control (CARE keV IQ level 50), and a sequential electrocardiogram-triggered scan was performed with an RR interval window fixed at 280 ms.

#### CT reconstructions of iodine maps

Iodine maps were reconstructed employing a quantitative kernel (Qr40), a section thickness of 1.0 mm at an increment of 0.5 mm. Iterative reconstruction was used at intermediate strength (Quantum Iterative Reconstruction level 3). A field of view of 200 mm, restricted to the heart, was applied, and the matrix size was set at 512^2^ pixels. All reconstructions were performed with the use of a proprietary offline image reconstruction platform (ReconCT, version 17.1.0.644; Siemens Healthineers).

### Image Interpretation

All images were independently reviewed by two blinded radiologists, one with board certification and 6 years of experience in cardiac imaging (M.T.H.), and the other with 5 years of training (G.T.), using a dedicated workstation (Syngo.Via, Siemens, software version VB70A, MM-reading workflow).

#### Assessment of subjective and objective image quality

For the subjective analysis of the images, both readers evaluated image noise on a 4-point scale as follows: 1, pronounced grainy appearance; 2, noticeable noise; 3, mild noise; and 4, minimal to no noise. The readers also assessed sharpness as: 1, poor definition of the left ventricular endocardial border and blurred edges; 4, excellent distinction with clear contours. Finally, overall image quality was rated as: 1, poor; 2, fair; 3, good; and 4, excellent.

For the objective assessment of image quality, regions of interest (ROI) with a minimum area of 60 mm² were manually placed in the mid-left ventricular septum and within the left ventricular blood pool. Measurements were repeated five times to ensure reliability, and the mean values were used for further analysis. For the repetition of measurements, consecutive axial images were used. Areas of fibrosis were carefully avoided to discount signal inhomogeneity. Image noise was defined as the standard deviation (SD) of the average CT number (in Hounsfield units, HU) within the blood pool. The CNR was calculated using the following formula: CNR = (HU blood pool − HU myocardium)/SD blood pool.

#### Diagnostic accuracy for myocardial LE detection

Iodine maps were evaluated for the presence of LE on a per-segment basis using the American Heart Association 17-segment model [22]. The two readers were allowed to adjust the window level to optimize visualization of late enhancement and increase diagnostic confidence. Multiplanar reconstructions were freely performed. Late enhancement patterns were classified as subendocardial, subepicardial, mid-wall, patchy, or transmural. Additionally, a qualitative per-patient assessment was performed to classify LE as ischemic or non-ischemic. LGE-MRI served as the reference standard and was independently analyzed by an adjunct committee, involving a board-certified radiologist with extensive experience in cardiac imaging [TE]. Furthermore, the assessment of ischemic and non-ischemic cardiomyopathies was made based on LGE-MRI.

### Statistical analysis

IBM SPSS Statistics for Macintosh (version 29.0.2.0), R (version 4.4.1, https://www.R-project.org/), and Prism Graph Pad (version 10.3.1) were used for statistical analysis. To check for the assumption of normal distribution, the one-sample Shapiro–Wilk test was applied. Quantitative variables were expressed as numbers (percentage), while continuous variables were mean ± SD, or median [interquartile range], as appropriate. For the subjective image quality assessment, the Wilcoxon signed-rank test was used to compare the readers’ evaluations. Independent measurements for objective image quality were compared using a paired *t*-test. To assess the impact of patient characteristics on image quality, a multivariate linear regression model was performed with image quality as the dependent variable and age, gender, and BMI as independent variables. The model fit was evaluated using the coefficient of determination (*R*^2^). To assess the diagnostic accuracy of iodine maps for detecting late enhacement, the results of the CT readings were compared to LGE-MRI, which served as the reference standard, both on a per-segment and per-patient basis. Sensitivity, specificity, positive predictive value (PPV), negative predictive value (NPV), and diagnostic accuracy were calculated and presented with 95% confidence intervals (CI). Interobserver agreement was measured using Cohen’s kappa (κ) statistics, with values interpreted as follows: poor (< 0.00), slight (0.00–0.20), fair (0.21–0.40), moderate (0.41–0.60), substantial (0.61–0.80), and almost perfect (0.81–1.00) agreement. A two-tailed *p*-value of < 0.05 was considered statistically significant.

## Results

### Patients

**Fig. 1:**
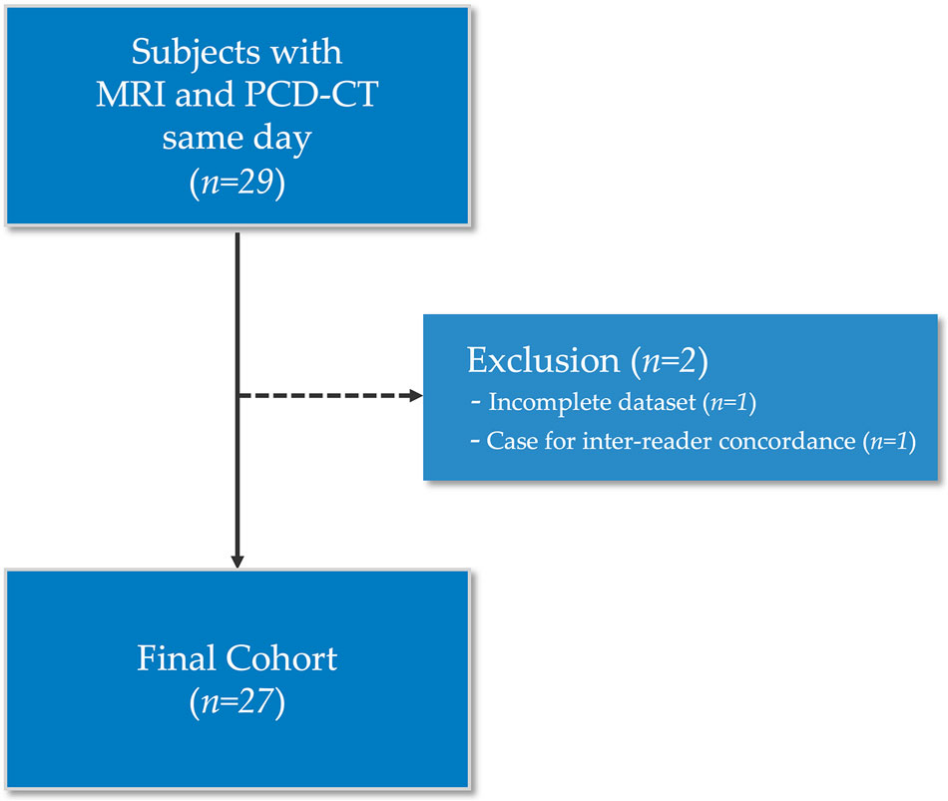
Study flow chart. After the exclusion of two subjects, the final cohort consisted of 27 participants, each receiving an MRI and PCD-CT scan with late enhacement on the same day

After excluding one patient due to an incomplete dataset and another one used as a training case to synchronize the interpretations between the two readers, the final study cohort comprised 27 patients (52% female; mean age 52.9 ± 17.2 years). Among these, 12/27 patients (44%) demonstrated positive LGE on MRI, with 3/27 (11%) having ischemic patterns and 9/27 (33%) showing non-ischemic patterns of enhancement. Additionally, 87 out of 459 myocardial segments (19%) were found to have positive LGE. No patient presented with myocardial edema. A patient flow chart is provided in Fig. 1, while the detailed characteristics are summarized in Table 1.

**Table 1:** Baseline characteristics

Characteristics	All patients (*n* = *27*)
Age (years)	52.9 ± 17.2
Sex (female)	14/27 (52%)
Body mass index (kg/m^2^)	28.5 ‍± 5.4
Cardiovascular risk factors	
Smoking	1/27 (4%)
Hypertension	18/27 (67%)
Dyslipidemia	10/27 (37%)
Diabetes	3/27 (11%)
Pulmonary hypertension	4/27 (15%)
Chronic heart failure	10/27 (37%)
History of CAD	6/27 (22%)
History of MI	3/27 (11%)
Valvular disease	11/27 (15%)
Radiation dose	
Calcium scoring CT	
CTDI_vol_ (mGy)	6.7 ± 2.9
DLP (mGy × cm)	100 ± 43
Coronary CT angiography	
CTDI_vol_ (mGy)	11.2 ± 10.5
DLP (mGy × cm)	173 ± 169
CT late enhancement	
CTDI_vol_ (mGy)	9.4 ± 8.6
DLP (mGy × cm)	110 ± 51
Patients with positive LGE	13/27 (48%)
Ischemic LGE	3/12 (25%)
Non-ischemic LGE	9/12 (75%)

Data expressed as mean ± SD or number and frequencies in parentheses

*CAD* coronary artery disease, *CTDIvol* computed tomography dose index volume, *DLP* dose length product, *LGE* late gadolinium enhancement

### Subjective and objective image quality

**Fig. 2:**
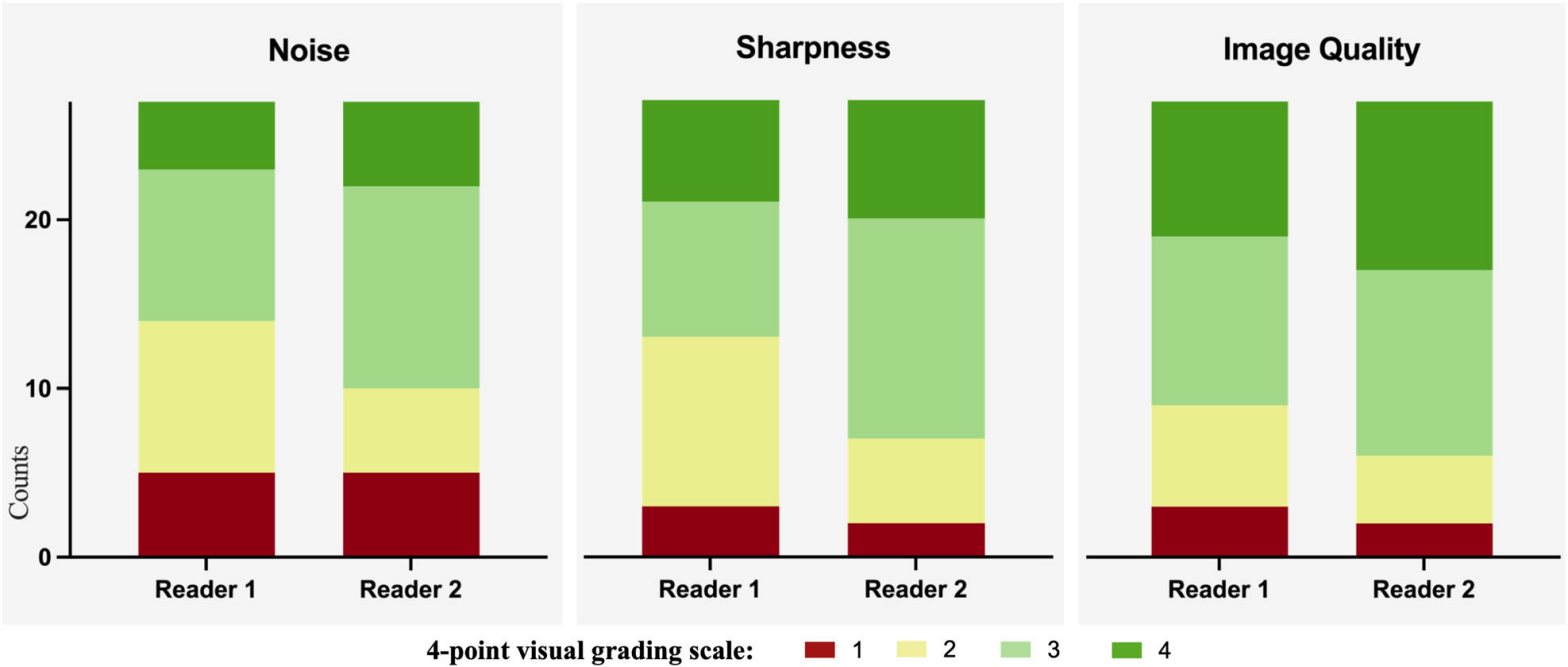
Stacked bar plots indicating the subjective image quality assessment of both readers regarding noise, sharpness, and overall image quality, using a 4-point scale

Iodine maps demonstrated overall good image quality, with no evidence of a difference between the two readers (median overall image quality 3 [2–4] vs 3 [3, 4]; *p* = 0.06). Both readers rated two cases as having poor image quality (Likert score 1), while reader 1 identified an additional case of poor image quality. Reader 1 rated 18/27 (66.7%) cases as either good or excellent (Likert score 3 or 4), compared to 21/27 (77.8%) cases rated by reader 2 (Fig. 2). Objective image quality metrics similarly showed no evidence of a difference: myocardial attenuation was 42.7 ± 15.7 HU for reader 1 and 43.3 ± 16.6 HU for reader 2 (*p* = 0.458), corresponding to a CNR of 3.4 ± 0.9 and 3.3 ± 1.1, respectively (*p* = 0.829). Detailed metrics for both subjective and objective image quality assessments are presented in Table 2. A multivariate regression analysis was conducted to explore the relationship between image quality and patient factors. BMI (*p* < 0.001), age (*p* = 0.002), and gender (*p* = 0.040) were significant predictors of image quality, with higher BMI, male gender, and older age correlating with lower image quality. Among these factors, BMI showed the strongest association (β = −0.1247), suggesting the highest impact on image quality degradation. The model explained 70.4% of the variance (*R*^2^ = 0.704), indicating a strong association. Details are provided in Supplementary Table 1.

**Table 2:** Subjective and objective image quality assessment

	Reader 1	Reader 2	*p*
Subjective image quality			
Noise	2 [2, 3]	3 [2, 3]	0.197
Sharpness	3 [2, 3]	3 [2–4]	0.109
Overall quality	3 [2–4]	3 [3, 4]	0.058
Objective image quality			
Myocardium attenuation (HU)	42.7 ± 15.7	43.3 ± 16.6	0.458
Blood pool attenuation (HU)	97.6 ± 21.8	93.9 ± 24.9	0.129
Image noise (HU)	16.7 ± 2.7	16.2 ± 3.8	0.390
CNR	3.4 ± 0.9	3.3 ± 1.1	0.829

Data expressed as median and interquartile range in square brackets, or mean ± SD

*HU* Hounsfield units

### Accuracy in myocardial LE detection

**Fig. 3:**
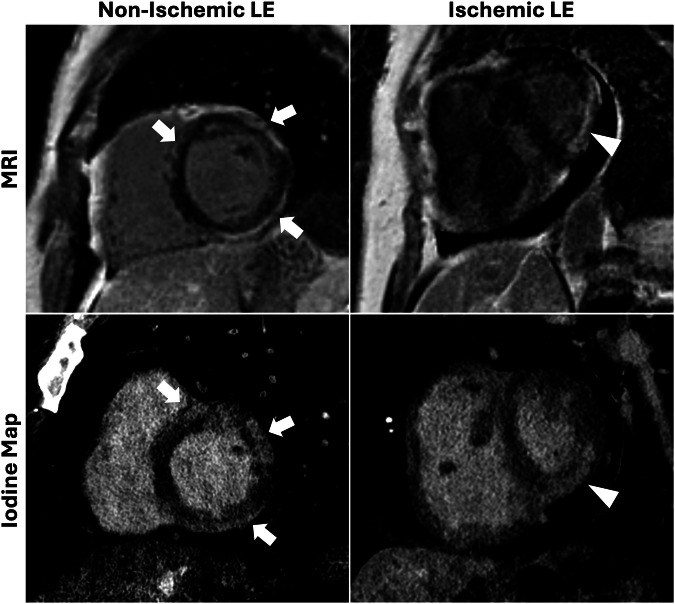
Non-ischemic and ischemic LE case examples. Top row: magnitude short-axis view late gadolinium enhancement images. Bottom row: short axis view of iodine maps generated from PCD-CT. The left column represents a case of a 50-year-old woman with a family history of arrhythmogenic ventricular cardiomyopathy, and subepicardial LE in the left ventricle (arrows), indicating areas of fibrous tissue. The right column is a 72-year-old man with a history of a myocardial infarction and previous stent implantation. A transmural LE is seen on the posterior basal and mid-ventricular wall (arrowhead), as well as a pericardial effusion. LE, late enhancement

In the per-patient analysis, reader 1 achieved a perfect sensitivity of 100%, with a specificity of 73.3%, while reader 2 showed a slightly lower sensitivity of 91.7%, with a specificity of 80.0%. Despite these differences, both readers demonstrated high diagnostic accuracy, achieving 85.2% each. An imaging example is given in Fig. 3. At the per-segment level, sensitivity values were 74.7% for reader 1 and 66.7% for reader 2, paired with very strong specificity rates of 94.9% and 96.4%, respectively. PPVs ranged from 77.4% to 81.1%, while NPVs remained consistently high at over 92% for both readers. Overall, per-segment accuracies reached 91.1% for reader 1 and 90.7% for reader 2 (Table 3). As a total, on MRI, a total of 87 segments were identified as positive for LE. In comparison, on CT, reader 1 detected 65 segments with LE, while reader 2 identified 58. Substantial inter-reader agreement was observed, with Cohen’s κ values of 0.70 per patient and 0.63 per segment.

**Table 3:** Diagnostic performance of iodine map from late PCD-CT scan compared to LGE MRI

	Reader 1	Reader 2
Per patient (*n* = 27)		
Sensitivity	100% (73.5–100)	91.7% (61.5–99.8)
Specificity	73.3% (44.9–92.2)	80.0% (51.9–95.7)
PPV	74.9% (56.4–87.4)	78.5% (56.7–91.1)
NPV	100% (71.5–100)	92.3% (64.4–98.8)
Accuracy	85.2% (66.3–96.8)	85.2% (66.3–95.8)
Per segment (*n* = 459)		
Sensitivity	74.7% (64.3–83.4)	66.7% (55.8–76.4)
Specificity	94.9% (92.1–96.9)	96.4% (93.9–98.0)
PPV	77.4% (68.5–84.4)	81.1% (71.6–88.0)
NPV	94.1% (91.8–95.8)	92.5% (90.2–94.3)
Accuracy	91.1% (88.1–93.5)	90.7% (87.8–93.2)

Data in parentheses represent the 95% CI range

*PPV* positive predictive value, *NPV* negative predictive value

### LE pattern evaluation

**Fig. 4:**
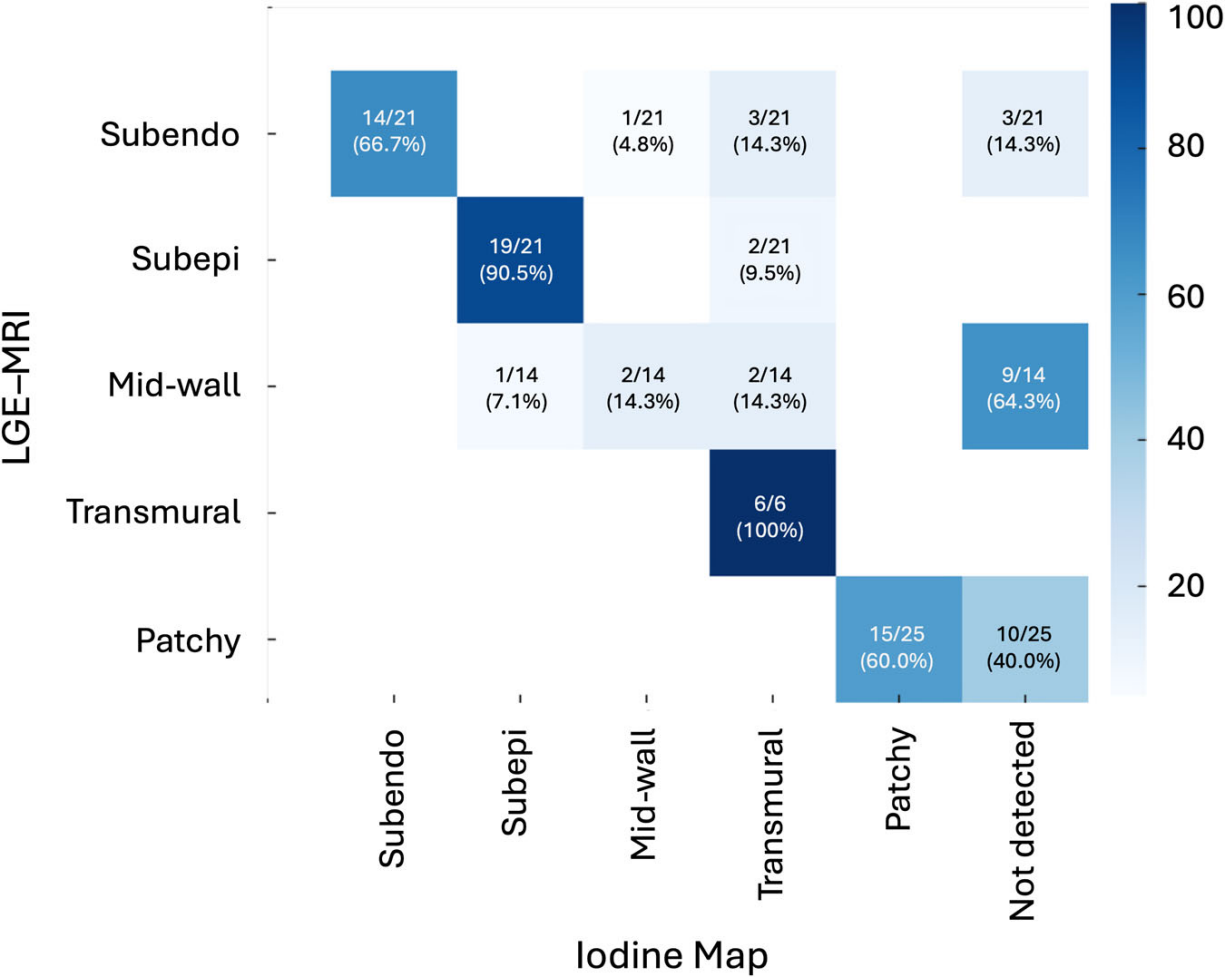
The heat map illustrates the segmental distribution of LE patterns as detected on iodine maps derived from PCD-CT. The most frequently observed late enhancement patterns include subepicardial (30.1%) and patchy fibrosis (26.2%), followed by subendocardial (21.5%), transmural (16.9%), and mid-wall (4.6%) involvement. Agreement between CT and MRI for segmental pattern identification was almost perfect (κ = 0.86, 95% CI: 0.75–0.97). LGE, late gadolinium enhancement; Subepi, subepicardial; Subendo, subendocardial

In total, ischemic patients (*n* = 3) had 27 affected segments on MRI, including 6 transmural and 21 subendocardial patterns, while non-ischemic patients (*n* = 9) had 60 affected segments, comprising 21 subepicardial, 25 patchy fibrosis, and 14 mid-wall patterns. For ischemic patterns, PCD-CT correctly identified 20 out of 27 (74%) segments. All 6 transmural segments were accurately detected. Among the 21 subendocardial segments, 3 were misclassified as transmural, 1 as mid-wall, and 3 were not detected, resulting in 7 total errors. For non-ischemic patterns, 36 out of 60 (60%) segments were correctly classified. Subepicardial LE was the most reliably detected pattern (19/21, 90%). Patchy fibrosis was detected in 5 out of 15 segments. Mid-wall pattern detection was poor, with only 2 out of 14 segments correctly classified, while 12 were misclassified or undetected. Among correctly identified segments, the most frequent LE patterns were subepicardial (*n* = 20, 30.1%), patchy fibrosis (*n* = 17, 26.2%), subendocardial (*n* = 14, 21.5%), transmural (*n* = 11, 16.9%), and mid-wall (*n* = 3, 4.6%). The agreement between CT and MRI for segmental LE pattern identification was almost perfect (κ = 0.86, 95% CI: 0.75–0.97). A detailed breakdown is provided in Fig. 4.

## Discussion

Our study aimed to evaluate the diagnostic accuracy of iodine maps generated by PCD-CT for detecting myocardial LE, using LGE-MRI as reference. The key findings of this investigation are: (i) iodine maps from PCD-CT provided good to excellent image quality in most cases; (ii) the technique demonstrated high diagnostic accuracy, 85.2% on per-patient and over 90% on per-segment level; and (iii) there was substantial inter-reader agreement, both at the patient and segment levels.

In our study, iodine maps from PCD-CT demonstrated high diagnostic accuracy for the detection of myocardial involvement using LIE-CT, with per-patient sensitivities of 100% (Reader 1) and 91.7% (Reader 2) and per-segment sensitivities of 74.7% and 66.7%, respectively. These findings outperform a prior study investigating LIE using dual-energy energy-integrating detector-CT and its accuracy in myocardial viability assessment (reported accuracy of 81% and sensitivity of 52%) [23]. The per-segment specificity of PCD-CT iodine maps was high (94.9% and 96.4%), indicating that false positives were rare. While this suggests a strong ability to confirm the absence of LE in non-involved segments, we acknowledge that specificity alone does not fully characterize the ability to rule out LE. The relatively high NPV observed in our study is influenced by the moderate to low prevalence of LGE-positive segments (19%), which can artificially inflate NPV values. On a per-segment level, sensitivity was lower (74.7% and 66.7%) than specificity. This finding is consistent with prior studies, where the detection of subtle or diffuse non-ischemic fibrosis remains challenging due to the inherent advantages of MRI in soft tissue contrast [24]. Nevertheless, the high per-patient sensitivity (100% and 91.7%) suggests that PCD-CT iodine maps are well-suited for identifying myocardial involvement at a clinically relevant level.

Given that clinical decision-making often occurs on a per-patient rather than per-segment basis, this finding reinforces the potential of PCD-CT iodine maps as a valuable tool for myocardial tissue characterization.

Mahnken et al reported strong correlations between LGE-MRI and LIE-CT in detecting acute myocardial infarction, with excellent agreement for enhancement size and location [25]. Our results confirm the capability of PCD-CT to provide a reliable alternative to MRI, particularly in patients with contraindications to MRI, or those unable to tolerate the examination. However, regardless of scanner technology, the accuracy of PCD-CT for detecting LIE remains inferior to that of LGE-MRI, emphasizing the inherent advantages of MRI in soft tissue contrast and scar detectability. The per-segment sensitivities observed in our study are more confirmatory than those reported in some dual-energy CT studies, which found strong agreement with LGE-MRI for segmental detection [24]. However, specificity reported in our study (94.9% and 96.4%) suggests that PCD-CT is highly accurate in ruling out LE. The difference in sensitivity can likely be attributed to the difficulty in detecting diffuse or subtle non-ischemic fibrosis, where MRI’s superior soft tissue contrast has an advantage [26]. However, ECV computation using CT can effectively diagnose diffuse myocardial fibrosis [27]. Therefore, the routine use of iodine maps could be advantageous, as it allows for simultaneous ECV computation and LE detection [20, 21].

Inter-reader agreement was substantial, consistent, or slightly inferior, to previous studies that report high inter-reader agreement in LIE-CT. Chang et al found excellent agreement (κ = 0.97) for detecting LIE using DECT [28]. Although the reported kappa values are lower, they still reflect substantial reliability between readers, suggesting that PCD-CT iodine maps can be interpreted consistently by different radiologists. The moderate variability observed in this study could be due to the subjective nature of subtle LIE interpretation, particularly in non-ischemic cases. While PCD-CT demonstrated excellent accuracy in detecting ischemic LE, particularly transmural patterns, non-ischemic patterns, especially mid-wall fibrosis or subtle, patchy fibrosis, were more frequently missed; additionally, the overall lower number of LE-positive segments detected on CT compared to MRI suggests a reduced sensitivity for subtle myocardial disease. Further standardization of reading protocols and training, or machine learning applications, may help improve consistency in future studies.

Subjective image quality was rated good to excellent in most cases, with no evidence of a difference between the two readers. This aligns with other reports on the performance of advanced CT technologies, including PCD-CT, which have demonstrated improvements in image quality by reducing artifacts such as beam hardening and blooming [29–31]. However, one of the persistent challenges for LIE-CT is the relatively lower CNR compared to MRI [25]. As mentioned in previous literature, LGE provides superior soft tissue contrast, which is particularly important for visualizing small or diffuse areas of fibrosis. Notably, our analysis showed that higher BMI was associated with lower image quality, suggesting that an increased iodine dose or optimized radiation settings may help mitigate this limitation. Our study confirms that while PCD-CT iodine maps provide high diagnostic accuracy, further optimization of CNR is necessary, potentially through the employment of advanced denoising algorithms or artificial boosting of the iodine signal. The application of spectral imaging and material decomposition in PCD-CT plays a critical role in improving image quality, but future innovations may focus on enhancing the differentiation between normal and fibrotic myocardium, e.g., for the assessment of myocardial viability. This may pave the way for PCD-CT to become a one-stop shop strategy to evaluate coronary arteries and the myocardium simultaneously. Such an approach may be particularly useful in patients undergoing pre-procedural assessment for structural heart disease, those with suspected ischemic or non-ischemic cardiomyopathy who cannot undergo MRI, or in emergency settings where rapid, comprehensive cardiac assessment is needed [32].

Despite the promising results, our study has some limitations. First, the small sample size of 27 patients limits the generalizability of the findings. Larger studies are needed to provide confirmatory research. Second, as the accuracy of ECV computation has already been investigated in a prior study of that prospective cohort, we deliberately avoided redundant reporting. Nevertheless, future research in an independent cohort is warranted to assess the combined value of visual LE detection and quantitative ECV computation, potentially enhancing the comprehensiveness of myocardial tissue characterization in a “one-stop-shop” approach. Third, while LGE-MRI was used as the established reference standard, no correlation to histology was performed. Fourth, this was a single-center study, which may limit the external validity of the results. Lastly, considering patients with contraindications to MRI, such as specific pacemakers, might greatly benefit from a LIE-CT assessment, but they did not consist of our study population and merit further investigation.

To conclude, iodine maps from PCD-CT demonstrate high diagnostic accuracy for assessing myocardial late enhancement, with substantial inter-reader agreement. These findings suggest that PCD-CT, with its inherent spectral capabilities, may serve as a valuable alternative to LGE-MRI in selected cases.

## Supplementary information

ELECTRONIC SUPPLEMENTARY MATERIAL

## Abbreviations

CI: Confidence intervals
CNR: Contrast-to-noise ratio
ECV: Extracellular volume
LE: Late enhancement
LGE: Late gadolinium enhancement
LIE: Late iodine enhancement
NPV: Negative predictive value
PCD-CT: Photon-counting detector CT
PPV: Positive predictive value
