# Supplementary material for: Accuracy of photon-counting detector CT-based iodine maps for myocardial late enhancement detection
Source: Eur Radiol. 2025 May 1;35(11):7074–83. doi: 10.1007/s00330-025-11622-0 (PMC12559134; doi:10.1007/s00330-025-11622-0)
Supplement: Supplementary file 1 — ELECTRONIC SUPPLEMENTARY MATERIAL [file 330_2025_11622_MOESM1_ESM.pdf]

# Accuracy of Photon-Counting Detector CT-based Iodine Maps for Myocardial Late Enhancement Detection

## ELECTRONIC SUPPLEMENTARY MATERIAL

**Supplementary Table 1.** Multivariate regression analysis of factors influencing image quality.

| Predictor | Coefficient (β) | Standard Error | t-value | p-value |
|---|---|---|---|---|
| Age (years) | -0.0227 | 0.007 | -3.444 | **0.002** |
| Gender (male) | -0.5037 | 0.231 | -2.183 | **0.040** |
| BMI (kg/m²) | -0.1247 | 0.022 | -5.753 | **<0.001** |
| Intercept | 7.879 | 0.728 | 10.830 | **<0.001** |
| Model Fit ($R^2$) | **0.704** | | | |

Note.—The table presents the results of a multivariate linear regression model assessing the association between image quality and patient characteristics. BMI and age were significantly associated with lower image quality, while male gender was also a significant predictor of lower image quality. R-squared ($R^2$) represents the proportion of variance explained by the model. Bold p-values indicate statistical significance at $p < 0.05$.
Abbreviations: BMI, body mass index; $R^2$, coefficient of determination.
